# Letter to the Editor: Virtual Reality in Facilitating Interaction with Police During Routine Traffic Stops for Persons with Autism

**DOI:** 10.1007/s10803-024-06647-1

**Published:** 2024-11-22

**Authors:** Anders Dechsling

**Affiliations:** Department of Education, ICT and Learning, Østfold University College, Halden, Norway

Dear Editor.

Recently, there has been an increased focus on driving skills as an important accomplishment for autistic individuals, and thus the driving license and the ability to go around in traffic as an important measure for equality of opportunity. As for autistic drivers, there is an increased chance of being stopped by police officers in traffic (Grindle et al., 2024). Gleeson et al., (2024) present the need for training both the autistic drivers and the police officers on the appropriate behavior during traffic stops.

In the article “Facilitating Interaction with Police During Routine Traffic Stops for Persons with ASD”, Grindle et al. (2024) demonstrate an excellent example of how to work with both autistic persons and police officers to ensure best possible outcomes. The authors describe the importance of driving for autistic individuals while also pointing to several challenges and the increased incidents of unwanted situations in traffic which may lead to interactions with police officers. In their paper, Grindle et al. (2024) describes the Blue Envelope program and a simulation training for both autistic individuals and police officers.

The Blue Envelope program is an initiative aiming to improve interactions between police officers and autistic individuals through enhanced mutual understanding, better communication, and thus safe and respectful encounters for both parties. Autistic drivers are provided with a distinctive blue envelope to hold their important documents (such as driver’s license, vehicle registration, and insurance). The blue envelope is easily recognizable and has an explanation of autism for the police officers, and useful information for communication and how both parties should act during the traffic stop (Grindle et al., 2024). In addition to the envelope as support material, both autistic drivers and police officers are offered routine traffic stops simulation training. There are several benefits of the traffic stop simulation such as an enhanced awareness and understanding of autism amongst police officers and thus a de-escalation of potential unwanted conflicts during traffic stops. Importantly, the Blue Envelope program and simulation training combined led to improvements in anxiety, comfort, and knowledge about future autism and police interactions – and as a general psychological tool for autistic drivers (Grindle et al., 2024).

Although there are several advantages of such programs and traffic stop simulation training, there are also challenges to be met. First, not all persons wish to flag their condition or diagnosis and therefore would like to keep it to themselves during a traffic stop situation. The other challenges are related to implementation and necessary training across states and police departments, and within the autism population. The training, of course, needs to show proper quality and effects, which are nicely demonstrated by Grindle et al. (2024). However, the challenges related to the wide implementation remain.

There are over 700 thousand police officers in USA (Korhonen, 2024), and probably over 2 million autistic persons with a driver’s license in USA. When considering the tremendous work completed in Grindle et al. (2024) with 48 autistic participants and two police officers per simulation, it seems clear that this type of work in a much broader context is resource demanding and time consuming. Although not everyone will need simulation training or a blue envelope, the increasing prevalence of autism suggest that these challenges will persist. I believe that there are measures that could be made to prevent the possibility of simulating traffic stops for autistic drivers and police officers to be discontinued due to implementation issues. I want to direct a focus towards the possibility of Virtual Reality (VR) technology as a possible tool for a wide implementation and for simulation training.

VR is a technology that allows presenters and users to display and experience digital realities and worlds. VR can be defined as technology that displays potential real-world like digital environments using visual and auditory stimuli through head-mounted displays (HMD), different set-ups using projectors, or desktop computer or tablet devices, with a possibility for the users to interact with the virtual environment. VR has received a lot of attention within autism research in recent years (Dechsling et al., 2022) and has been considered to have several advantages as a supplemental tool for assessment and interventions for autistic individuals. For instance, VR offers the ability to create a safe and controlled environment, eliminating potential real-life unwanted consequences of social interactions during practice (Dechsling et al., 2022). Furthermore, VR offers the possibility to quickly make transitions between various locations and scenarios for simulation (Dechsling et al., 2022), and the possibility to adjust the intensity of features within VR to avoid sensory overload (Skjoldborg et al., 2022). More importantly, VR seems to be an acceptable tool when evaluated by autistic individuals (Newbutt et al., 2020) even though individual considerations needs to be made (Dechsling et al., 2020). However, although the reiteration of suggestions that VR is promising in improving various skills, there are still to date no decisive evidence on the general effectiveness of using VR as a tool.

Nevertheless, the possible advantages of VR appear as real. I believe that applying VR to make traffic stop simulation and practice more available is a feasible path – for both autistic individuals and police officers. Among few areas, VR has shown effectiveness for driving skills (Carnett et al., 2023) and overcoming barriers related to phobia (Maskey et al., 2019). VR driving simulation is a feasible way of enhancing driver training as it offers a versatile, safe, and cost-effective solution for preparing drivers before entering the roads. Simulating routine traffic stops, and for instance scenarios involving the Blue Envelope, should be feasible to implement as an additional application in driving simulations – or as a standalone VR-application. The safe controlled environment can be beneficial to reduce potential anxiety and prepare for real-life encounters.

In addition, VR has been used and found useful to demonstrate and raise awareness of how the perspective of an autistic individual may appear (Koniou et al., 2023). I would suggest that there is a high probability that similar demonstrations would be useful also for service personnel and law enforcers, although it is necessary to also be aware of the heterogeneity in the autism population. Such demonstrations can be used to simulate and mimic the sensory or communication challenges an autistic individual might experience, and thus provide the officers better insights and understanding. These examples demonstrate the feasibility of VR to continue approaching both parties in meeting the challenges that may occur during traffic stops.

In conclusion, integrating VR technology to simulate and practice traffic stops for autistic drivers and police officers could solve several challenges associated with training, awareness, and practical application. VR could potentially assist in the making traffic stop simulation programs more effective and widespread. As VR technology continues to advance, its potential in assisting the routine traffic stop simulations and similar initiatives will most likely expand, and thus widen the possibilities to disseminate inclusive and compassionate public safety practices. Furthermore, VR can be used as a cost-effective measure towards a standardization of the simulation training across different law enforcement agencies, which could ensure a respectful consistency in how police officers are educated about approaching and cooperating with autistic drivers, and vice versa. In addition, VR allows for ongoing education and for brushing-up on knowledge and skills so that both autistic drivers and police officers can regularly engage with new scenarios and updates. The nice demonstration of Grindle et al. (2024) show the positive effects of simulation training, and I suggest that VR is a tool that can potentially expand these nice results in the future.
